# Postural Adaptations of the Spinopelvic Parameters in the Sagittal Plane Induced by the Use of High-Heeled Shoes in a Sample of Young Healthy Female Subjects: A Pilot Study

**DOI:** 10.7759/cureus.83768

**Published:** 2025-05-09

**Authors:** Saverio Colonna, Corrado Borghi, Eleonora Canova

**Affiliations:** 1 Rehabilitation Medicine, Spine Center, Bologna, ITA; 2 Research and Development, Osteopathic Spine Center Education, Bologna, ITA; 3 Research, Spine Center, Bologna, ITA

**Keywords:** anterior pelvic tilt, high heels, low back pain, lumbar lordotic angle, postural adaptations, raster-stereography, spinopelvic profile

## Abstract

This pilot study aimed to investigate postural adaptations in spinopelvic parameters on the sagittal plane following the use of high-heeled shoes over a prolonged period in a population of healthy young women. Thirty-four female participants were assessed using rasterstereography at three time points: standing barefoot (T1), immediately after wearing their own high-heeled shoes (T2), and after two consecutive hours of standing or walking while wearing the shoes (T3). The evaluation focused on trunk inclination, pelvic tilt, lumbar lordotic angle, and thoracic kyphotic angle. Participants were also stratified into two subgroups based on self-reported history of low back pain (LBP) during high-heel use: 13 symptomatic and 21 asymptomatic individuals.

The statistical analysis, conducted using both parametric and non-parametric tests due to the borderline normality of the data, revealed no significant postural changes between T2 and T3, nor any relevant differences between the two subgroups. Only a modest variation in the kyphotic angle was observed between T1 and T3, which may not be clinically relevant. Notably, none of the participants reported pain after the two-hour protocol.

These findings suggest that, in inexperienced users, high-heeled shoes do not induce significant spinopelvic compensations or acute lumbar discomfort following moderate-duration use. As this is the first study to evaluate postural outcomes after sustained use rather than immediate heel elevation, further research with larger and more diverse samples is warranted to better elucidate the potential role of heel-induced adaptations in the etiology of LBP.

## Introduction

High-heeled footwear is commonly worn to enhance height, elegance, and physical attractiveness. Nonetheless, several authors have suggested that habitual use of high heels may increase the risk of developing low back pain in women [1]. The reported incidence of such pain has led to investigations focusing on spinopelvic alignment alterations as potential contributing factors.

Studies examining this aspect sought to uncover compensatory and adaptive structural changes as potential contributors to lower back pain. The conclusions from these works, however, are controversial. Several studies [2,3] have reported that wearing high-heeled shoes significantly increases lumbar lordosis (LL), which may in turn elevate mechanical loading and contribute to degenerative changes in surrounding soft tissues [4]. Conversely, other investigations [5,6] have observed a decrease in LL, accompanied by a compensatory rise in erector spinae muscle activity [7]. Findings from other studies have indicated that heel elevation does not produce significant changes in LL among the subjects assessed [8-10]. Instead, raising the heel seemed to acutely modify the balance on the sagittal plane; for some authors as the height of the heel increases, the anterior inclination of the trunk increases, according to others [11] the inclination of the trunk and pelvis is reduced, while others [9] have reported no change in trunk and pelvic inclination.

Oliveira Pezzan et al. [7] reported distinct postural responses between habitual and non-habitual high-heel users: an increase in pelvic anteversion and LL was observed exclusively in frequent users, while non-frequent users exhibited a reduction in both pelvic tilt and lordosis.

All the studies present in the literature take into consideration only acute changes, i.e. immediately after the raising of the heel. In reality, lumbar pain symptoms, linked to an upright posture, appear after maintaining the posture for some time. For instance, in a 2008 study [12], 65% of previously asymptomatic participants developed low back pain (LBP) in response to a two-hour standing protocol.

The aim of this study is to: (1) evaluate the postural changes of the pelvis and spine in a group of young, healthy female subjects after wearing their high-heeled shoes for at least two hours; and (2) compare whether there are differences between subjects who report experiencing pain and those who do not when wearing high-heeled shoes.

## Materials and methods

The hypothesis was to assess whether the spinopelvic profile changes after muscular fatigue due to two hours of standing posture. This hypothesis will be verified through a monocentric cross-sectional study conducted at the Spine Center, Bologna, Italy.

The participants were female students voluntarily recruited from the Osteopathic Spine Center Education (OSCE) School of Osteopathy in Bologna, Italy, through announcements made during classes and posted on the school’s bulletin board. All students who expressed interest and met the inclusion criteria were enrolled. Individuals were excluded if they had structural (e.g., severe scoliosis, spondylolisthesis) or neurological conditions (e.g., peripheral neuropathies, balance disorders) that could impair their ability to maintain an upright posture for at least two consecutive hours while wearing heels of 7 cm or higher.

Participants provided information on age, height, weight, and shoe size. A questionnaire was also administered to document the type of high-heeled shoes worn and the frequency of their use. Based on the medical history data, participants were classified as "inexperienced" high-heel users, according to the criteria of Oliveira Pezzan et al. [7], since they reported wearing high heels less than twice a week and/or for less than three hours per week.

The study protocol adhered to the ethical standards of the Helsinki Declaration, ClinicalTrials registration number NCT05593991 and the Institutional Review Board (IRB) of Manus Sapiens issued approval No. 002-2023.

The raster-stereography method (Formetric 4D®; Diers International GmbH, Schlangenbad, Germany), a non-invasive, radiation-free optical measurement system used to assess spinal posture and pelvic alignment, was used to evaluate the spinopelvic parameters. This is a commercially available device.

Participants underwent three measurements of spinopelvic parameters: barefoot, immediately after donning the shoes, and after wearing the shoes for two hours. A two-hour period was chosen because a study by Nelson-Wong et al. [12] reported clinically significant LBP in response to a two-hour standing protocol. However, the article makes no reference to the type of shoes.

**Figure 1:**
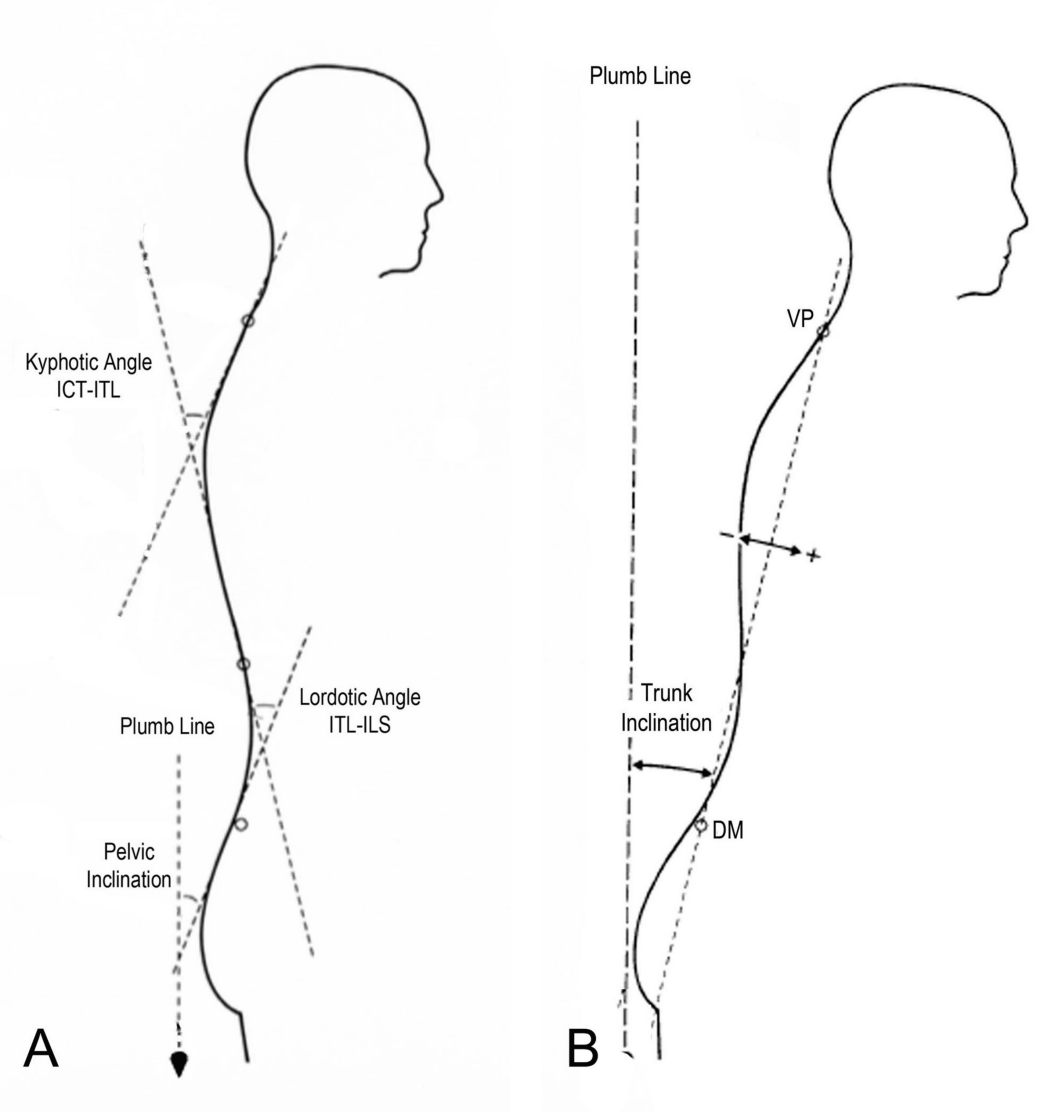
Spinopelvic rasterstereographic parameters collected A: Pelvic inclination angle (PI), lordotic angle (LA) and kyphotic angle (KA); B: antero-posterior trunk flexion (trunk inclination (TI)) ILS: lumbosacral junction, ITL: thoracolumbar junction, ICT: cervicothoracic junction, VP: prominent cervical vertebra, DM: two dimples Reproduced from Colonna et al. [10] under a Creative Commons Attribution License (CC BY).

Data collected are shown in Figure 1A: pelvic inclination angle (PI), the angle spanned by the vertical and the tangent lumbosacral junction (ILS), lordotic angle (LA) thoracolumbar junction (ITL)-ILS, measured between the tangents of the ITL, and the ILS, kyphotic angle (KA) cervicothoracic junction (ICT)-ITL, measured between the tangents of the ICT and the ITL; and in Figure 1B: antero-posterior trunk flexion (trunk inclination (TI)) measured as the angle between the plumb line and the line passing through the prominent cervical vertebra (VP) to the line that connects the two dimples (DM).

Evaluation protocol

Participants were prepared to ensure full visibility of the spine from the hairline to the sacrum. Data collection was performed while subjects stood in a relaxed posture, with knees fully extended and arms resting naturally alongside the hips. To standardize positioning, adhesive tape was placed on the floor as a reference for foot placement.

Measures were conducted in the following sequence: (1) barefoot, in a neutral position (T1); (2) immediately after donning the shoes (T2); and (3) after two hours of standing or walking with the shoes, without sitting (T3).

Participants at T2 and T3 wore their own pair of heels. The only request was to wear a shoe with a heel height greater than 7.6 cm (3 inches), a threshold beyond which a heel is considered high by the American Podiatric Association [13].

The Numeric Pain Scale (NPS) was used to quantify the presence of pain after the two hours of the experiment. Rasterstereographic assessments were consistently conducted by the same two experienced operators. Subsequently, a third operator transcribed the results into an Excel spreadsheet and carried out the statistical analysis.

Statistical methods

The Shapiro-Wilk test was used to assess the normality of the data distribution. If the assumption of normal distribution was met, postural changes on the sagittal plane induced by wearing heeled shoes for at least two hours were analyzed using repeated measures ANOVA and paired t-tests, testing the following hypotheses:

H₀: X_T1 = X_T2; H₁: X_T1 ≠ X_T2 H₀: X_T2 = X_T3; H₁: X_T2 ≠ X_T3 H₀: X_T1 = X_T3; H₁: X_T1 ≠ X_T3

In the case of a statistically significant ANOVA result, Bonferroni post hoc tests were conducted. If the normality assumption was violated, the Friedman test was used to assess differences across T1, T2, and T3, and the Wilcoxon signed-rank test was applied to compare T2 and T3. In the event of a significant Friedman result, the Dunn-Bonferroni post hoc test was performed.

The logistic regression model included individual characteristics such as age, height, weight, and shoe size, along with postural parameters, to estimate the probability of developing LBP.

\[ \Pr\{LBP\} = \frac{e^{\beta_0 + \beta_1 \cdot \text{Age} + \beta_2 \cdot \text{Weight} + \beta_3 \cdot \text{Height} + \beta_4 \cdot \text{Shoe Size} + \beta_5 \cdot X_{T1} + \beta_6 \cdot (X_{T3} - X_{T2})}}{1 + e^{\beta_0 + \beta_1 \cdot \text{Age} + \beta_2 \cdot \text{Weight} + \beta_3 \cdot \text{Height} + \beta_4 \cdot \text{Shoe Size} + \beta_5 \cdot X_{T1} + \beta_6 \cdot (X_{T3} - X_{T2})}} \] For each variable, the statistical significance of the β₆ coefficient associated with the variation in posture was tested. In the case of statistical significance, the interpretation of the identified coefficient was provided. An estimate of the effect size (Cohen’s d and r) was also reported for each variable. Significance was accepted for p ≤ 0.05.

## Results

Thirty-four females took part in the study (age 21.6±1.9; height 165.9±5.9 cm; weight 58.1±6.4 kg; shoe size 38.2±1.3). Thirteen of them had experienced LBP in the past while using high-heeled shoes (Group S - symptomatic), and 21 had not (Group A - asymptomatic). The average height of the heel was 9.1±1.7 cm. No subject complained of pain after the two-hour analysis.

The evaluation of the data obtained using the Shapiro-Wilk test highlighted that in only one of the parameters detected (LA at T1) there was acceptance of normally distribution (p >0.05); anyway, by comparing graphically the parameters investigated with the normal, it is possible to note, for most of the variables, a bell-shaped distribution with a slightly asymmetric unimodal trend. For this reason and aware that the underlying error we would encounter using only the data obtained as not normally distributed, it was decided to continue to treat the data, in both ways, as if they were all normally and also as if they were not normally distributed.

Table 1 presents the data as mean and standard deviation for the measurements at T1, T2, and T3. The table also reports the results of the ANOVA test (p-value and F-value) considering all three time points. The subsequent columns show the comparison between T2 and T3, which was the focus of this study, including the p-value, t-value, and effect size (d-Cohen).

**Table 1:** Parameters recorded in degrees (°) by rasterstereography in 34 participants Mean and standard deviation at T1, T2, and T3; p-value and F-value from ANOVA; p-value, t-value, and effect size (Cohen’s d) from Student’s t-test comparing T2 and T3. This table presents the results of the parametric analysis. * statistical significance at p ≤ 0.05. TI: trunk inclination, PI: pelvic inclination, LA: lordotic angle, KA: kyphotic angle

parameter	T1 (mean ± SD)	T2 (mean ± SD)	T3 (mean ± SD)	p value ANOVA	F value	P value (T2 vs T3)	T value (T2 vs T3)	effect size (d Cohen)
TI (°)	1.74±2.7	2.16±2.9	1.80±2.4	0.24	1.44	0.22	1.24	0.21
PI (°)	23.84±5.2	22.72±6.1	22.84±5.3	0.22	1.53	0.09	1.73	0.03
LA (°)	46.45±8.2	45.72±9.3	45.43±8.6	0.27	1.3	0.44	0.66	0.08
KA (°)	47.90±7.9	48.72±8.3	50.66±7.5	0.01*	4.87	0.09	-1.73	0.3

As shown in Table 1, none of the parameters reached the predefined level of statistical significance, except for the variance analysis of KA (p = 0.01). The Bonferroni post hoc analysis revealed that, for the KA parameter, only the comparison between T1 and T3 reached statistical significance (p = 0.021, t = -2.87).

Based on the statistical analyses, the null hypotheses (H₀) comparing T1 vs. T2, T2 vs. T3, and T1 vs. T3 were not rejected for any of the investigated parameters, with the exception of the KA, where a significant difference was observed between T1 and T3.

Table 2 reports the data treated as non-parametric, expressed as median and interquartile range for the measurements at T1, T2, and T3, along with the results of the Friedman test and the chi-square value. The final three columns present the results of the Wilcoxon signed-rank test (W and Z values) and the effect size (r-Cohen) for the comparison between T2 and T3, which represents the objective of this study.

**Table 2:** Parameters recorded in degrees (°) by rasterstereography in 34 participants, treated as non-parametric data. Values are expressed as median and interquartile range at T1, T2, and T3; results of the Friedman test and chi-square values are reported. The final three columns show the results of the Wilcoxon signed-rank test (W and Z values) and the effect size (Cohen’s r) for the comparison between T2 and T3. TI: trunk inclination, PI: pelvic inclination, LA: lordotic angle, KA: kyphotic angle

parameter	T2_A (mean ± SD)	T2_S (mean ± SD)	T2-p value	T2-t value	T3_A (mean ± SD)	T3_S (mean ± SD)	T3-p value	T3-t value
TI (°)	3.62±3.73	1.26±2	0.05	2.11	2.62±3.1	1.29±1.8	0.17	1.41
PI (°)	23.33±5.72	22.35±6.65	0.66	0.44	23.28±5.18	22.57±5.6	0.71	0.37
LA (°)	46.29±9.59	45.37±9.61	0.78	0.27	46.05±9.59	45.05±8.52	0.75	0.32
KA (°)	48.51±7.44	48.85±9.2	0.91	0.11	50.08±6.8	51.01±8.24	0.73	-0.34

Similarly, the non-parametric analyses did not provide sufficient evidence to reject the null hypotheses for any of the comparisons between T1, T2, and T3, confirming the findings obtained with the parametric approach.

Using the t-test and also the Wilcoxon test, no significant differences can be seen between the T2 and T3 samples in the overall sample; the same within each of the symptomatic and asymptomatic subgroups.

The comparison between group S and group A does not highlight statistical differences in age, height and weight. Table 3 presents the statistical comparison, using the independent samples Student’s t-test, of the rasterstereographic measurements from the T2 and T3 acquisitions between the S and A groups, treated as parametric data. As shown, none of the parameters reached the predefined level of statistical significance (p ≤ 0.05).

**Table 3:** Rasterstereographic parameters (TI, PI, LA, KA) from the second (T2) and third (T3) acquisitions Asymptomatic (A) and symptomatic (S) groups compared as parametric data using the independent samples Student’s t-test. TI: trunk inclination, PI: pelvic inclination, LA: lordotic angle, KA: kyphotic angle

parameter	T2_A_mean (SD)	T2_S_mean (SD)	T2-p value	T2-t value	T3_A_mean (SD)	T3_S_mean (SD)	T3-p value	T3-t value
TI (°)	3.62±3.73	1.26±2	0.05	2.11	2.62±3.1	1.29±1.8	0.17	1.41
PI (°)	23.33±5.72	22.35±6.65	0.66	0.44	23.28±5.18	22.57±5.6	0.71	0.37
LA (°)	46.29±9.59	45.37±9.61	0.78	0.27	46.05±9.59	45.05±8.52	0.75	0.32
KA (°)	48.51±7.44	48.85±9.2	0.91	0.11	50.08±6.8	51.01±8.24	0.73	-0.34

## Discussion

In the literature, many studies have addressed the problem of back pain linked to high-heeled shoes. Analysis of the effect of high heels on the pelvis and spine has yielded conflicting results [1-12].

A possible explanation of this controversy is that the postural modifications determined by the heels are highly subjective and therefore variable. Another explanation could lie in the choice of the condition investigated. In fact, all assessments, to the knowledge of the authors, were conducted in the acute phase, i.e. after a very short time after wearing high-heeled shoes. However, it is plausible that time is relevant: maintaining a standing posture, which adopts compensatory mechanisms immediately after heel elevation, as reported in some studies with shoes [3,8,9,14] or artificial devices [10,11,15], may lead to the onset of muscle fatigue over time.

To the authors' knowledge, only one work reported a correlation between high heels and LBP (associated with lower limb pain) that was not in the acute phase [16]. This correlation was identified after 20 minutes of high heels use.

Anyway, it is actually conceivable that spinopelvic alterations may develop over time due to neuromuscular fatigue [17], prompting the decision to assess differences after a two-hour period.

Notably, none of the studies identified in the literature have documented lumbar symptoms immediately following heel elevation in subjects standing upright. Instead, a study by Nelson-Wong et al. [12], albeit not specific to high-heeled shoes, has reported clinically significant LBP in response to a two-hour standing protocol. While not all asymptomatic individuals experience LBP during prolonged standing, their transient LBP model has enabled the characterization of neuromuscular distinctions between those who develop LBP and those who do not. However, in our study, none of the subjects reported LBP after two hours of weight-bearing posture in their own shoes, with an average heel height of 9.1 cm.

It is noteworthy that, unlike the study by Nelson-Wong et al. [12], where subjects were required to maintain a static upright posture without necessarily wearing high-heeled shoes, our participants were allowed to stand still or walk (only prevented from sitting).

Comparing spinometric parameters of the pelvis and spine of the complete group of 34 subjects between T2 and T3, no significant differences were observed, regardless of whether the data were processed parametrically or non-parametrically, except for the comparison of the KA parameter, which the post hoc analysis highlights as significantly different only for the comparison between T1 and T3.

As stated in the introduction, the aim of the study was mainly to evaluate what changes after two hours of wearing heels, as the acute modification from the heel raise has already been addressed in a previous study [10] where we used a larger sample (48 female subjects and 49 male subjects).

Therefore all subjects managed not to significantly change their posture after the two hours of charging. The same applies when crossing the data of the two subgroups (21 group A and 13 group S); no significant differences can be appreciated by treating the data as normally distributed.

We processed the data both as if they were normally distributed and as if they were not normally distributed, because in our previous work [10], in which we used the same detection methodology, we encountered the same result: the Shapiro-Wilk test showed that some parameters met the normality condition while others did not. However, given the slightly asymmetric unimodal distribution, we preferred to use, in that study, only parametric tests.

In this work, as this is a pilot study, we chose to apply both parametric and non-parametric methods to minimize the risk of bias due to the improper use of statistical tests. Regarding the effect size, it can be observed that most of the parameters analyzed, both as parametric data using Cohen’s d (Table 1) and as non-parametric data using Cohen’s r (Table 2), did not reach an acceptable level, a result likely attributable to the small sample size used. While standing posture, irrespective of shoe type, appears to be associated with a form of LBP [12,18], the same cannot be said for high-heeled shoes.

The challenge in clearly establishing this association is evident in the most recent systematic review from 2023 on high heels and LBP [1]. The review included 11 studies, with nine confirming an association and two not. Upon closer examination of the studies, only two reported an association [19,20], two reported no association [21,22], and the conclusions of the other are not very clear [8,14,16,23-25].

Regarding this lack of clarity, for instance, one of the aforementioned articles [16] reported data on lower back pain and pain in the muscles of the lower limb after a specific period of time, precisely 20 minutes, of wearing high-heeled shoes. The results suggest a correlation between heel height and perceived lower back pain. However, it should be noted that the task assigned to the tested subjects was to walk on a treadmill at 4 km/h for 20 minutes while wearing shoes with a 5 cm stiletto heel. It is intuitive that the 5 cm stiletto heel, especially when used on a surface not typically associated with treadmill use, could cause lumbar pain that is not strictly related to the postural changes induced by the heel lift.

The results of this pilot study suggested a lack of association between high heels and significant spinopelvic changes, which hypothetically could be linked to LBP.

Limitations of the study

Since this is the first study investigating the effects of wearing high-heeled shoes for two hours on spinopelvic parameters, acquired through rasterstereography, it was classified as a pilot study.

A primary limitation of this pilot study is the overall small sample size, which reduces the statistical power of the analysis. This issue is particularly relevant in the symptomatic subgroup, which was numerically underrepresented compared to the asymptomatic group, limiting the reliability of between-group comparisons. Furthermore, the limited variability among participants - all young, healthy, and inexperienced high-heel users - restricts the generalizability of the findings to broader populations.

## Conclusions

As this is a pilot study, no definitive conclusions can be drawn regarding the long-term effects of high-heeled shoe use on sagittal spinopelvic alignment. However, the absence of significant postural alterations after two hours of continuous wear, along with the lack of reported low back pain among participants, suggests that in young, healthy, and inexperienced users, short-term use of high heels may not substantially influence spinopelvic posture or trigger lumbar discomfort.

Given the lack of comparable studies evaluating postural parameters after prolonged wear rather than immediate heel elevation, these findings provide a preliminary reference for future investigations. Larger-scale studies involving more diverse samples, including subjects of different ages, body types, habitual high-heel use patterns, and pre-existing musculoskeletal conditions, are warranted to confirm these results, better define individual susceptibility, and explore the potential mechanisms linking high-heeled footwear to low back pain and other postural complaints.
